# Laser-Induced Graphene Based Flexible Electronic Devices

**DOI:** 10.3390/bios12020055

**Published:** 2022-01-20

**Authors:** Hao Wang, Zifen Zhao, Panpan Liu, Xiaogang Guo

**Affiliations:** Institute of Advanced Structure Technology, Beijing Institute of Technology, Beijing 100081, China; 3120205861@bit.edu.cn (H.W.); 3220190384@bit.edu.cn (Z.Z.); 3220190288@bit.edu.cn (P.L.)

**Keywords:** laser-induced graphene (LIG), flexible electronics, biosensor, health monitoring, electrode

## Abstract

Since it was reported in 2014, laser-induced graphene (LIG) has received growing attention for its fast speed, non-mask, and low-cost customizable preparation, and has shown its potential in the fields of wearable electronics and biological sensors that require high flexibility and versatility. Laser-induced graphene has been successfully prepared on various substrates with contents from various carbon sources, e.g., from organic films, plants, textiles, and papers. This paper reviews the recent progress on the state-of-the-art preparations and applications of LIG including mechanical sensors, temperature and humidity sensors, electrochemical sensors, electrophysiological sensors, heaters, and actuators. The achievements of LIG based devices for detecting diverse bio-signal, serving as monitoring human motions, energy storage, and heaters are highlighted here, referring to the advantages of LIG in flexible designability, excellent electrical conductivity, and diverse choice of substrates. Finally, we provide some perspectives on the remaining challenges and opportunities of LIG.

## 1. Introduction

Advanced electronics with the combined attributes of multifunctionality, high integrability and flexibility are becoming increasingly important due to the development of wearable and flexible electronics. Tremendous efforts have been devoted toward pursuing the functional materials with an inherent flexible or stretchable property, or optimizing the geometrical configurations to meet the complex target shapes. Additionally, the fabrication of the highly conductive material in a fast, low-cost, efficient, and environmentally friendly method always stands out as the major requirement for flexible electronics. Recently, graphene, a two-dimensional material, has been of growing interest due to its excellent electrical conductivity, mechanical, optical, and thermal properties [1]. They have found their potentials in many fields ranging from wearable electronics, health monitors, motion captures, to soft robots. The graphene has been successfully fabricated through a variety of methods, including mechanical exfoliation [2], chemical vapor deposition (CVD) [3], and chemical reduction of graphene oxide (GO) [4,5]. Mechanical exfoliation can obtain larger sizes and high-quality graphene. However, this method is only suitable for scientific research, and its production efficiency is too low to be used in large-scale production. In addition, sonication assisted liquid-phase exfoliation can successfully exfoliate graphite in liquid environments by exploiting ultrasound to extract individual layers. This method can obtain high-quality graphene inks with a high production efficiency using the common equipment in labs. However, the production of graphene through this fabrication method produces a large amount of waste and requires long lasting sonication treatments [6]. CVD is one of the ideal methods for large-scale graphene production and can be used to prepare high-quality single or multi-layer graphene, nevertheless, it is difficult to popularize due to strict experimental conditions and multi manufacturing steps limitations. Chemical reduction of GO is a low-cost and high production efficiency method, but there are toxic gas and explosion hazards in the preparation process. However, the multi manufacturing steps, the sophisticated operation procedure, the cost-effective synthesis and patterning of carbon nanomaterials of these manufacturing as mentioned above techniques impose certain limitations on the practical applications, and it is still challenging to fabricate and pattern the graphene through an in situ, one-step and scalable approach. In contrast to chemical reduction of GO, irradiation of the GO film with an infrared laser inside an inexpensive commercially available LightScribe CD/DVD optical drive, reduces the GO to laser-scribed graphene [7]. Laser equipment has been popular in factories and laboratories, and it can be used for precisely and rapidly fabricating patterns for various functional applications. Laser direct write technology has been demonstrated as a reliable, mask-free and template-free method [8,9]. The researchers accidently discovered that the PI could be directly converted to porous graphene using an infrared CO_2_ laser while cutting PI films in 2014 [10]. The technology of photothermally converting organic films to continuous 3D porous graphene structures by pulsed laser irradiation under ambient air is known as the formation procedure of laser-induced graphene (LIG). This method has a high production efficiency, but only generates multilayer graphene. The underlying mechanisms mainly involve the carbonization occurring on the surface of the PI substrates under laser scribing [11,12]. The sharp rise in the localized temperature due to the laser irradiation breaks the C–O, C=O, and N–O bond and leads to the recombination of C and N atoms [13]. In this method, any desired complex graphene pattern can be directly formed on the carbon sources films, facilitating the fabrication procedures for the individually customized electrical devices. Ascribing to its advantages of the mask-free and visual fabrication process, numerous efforts have been devoted to improving the preparation process and enriching the accessible materials for LIG. Recently, LIG have been successfully prepared by a variety of laser sources according to the properties of the initial carbon precursor. Energy storage, catalysis, sensing and biomedical applications also have been realized by controlling the microstructure, doping amount and type, as well as post-deposition methods [14,15]. Recently, the visible and ultraviolet (UV) lasers also have been demonstrated in successfully preparing LIG on PI substrate [16,17,18,19,20]. In addition to pulsed lasers, continuous wave (CW) laser beam can also be used to fabricate LIG [21]. LIG fabricated by CW lasers exhibit optical anisotropy. The detected anisotropy is due to the specific orientation of the graphene-containing formations relative to the PI/LIG interface during the LIG formation. Furthermore, a variety of natural and synthetic materials, ranging from plants [22,23,24], textiles [24,25,26,27], papers [28,29,30] to other organic films [17,27,31,32,33,34], are experimentally demonstrated in serving as the carbon source to form LIG. Meanwhile, the conductivity, electrochemical performance [24,27,35,36], biocompatibility [37,38], and hydrophobicity [39,40,41] of LIG also have been systematically studied. A variety of LIG devices have been developed, including sensors [14,15,16,24,25,26,27,28,29,30,31,32,33,34,35,36,37,38,39,40,41,42], supercapacitors [17,43,44,45,46,47,48,49,50,51,52,53,54,55], nanogenerators [54,55,56,57,58,59,60,61], heaters [28,29,62,63,64,65,66,67], catalysts [68,69,70,71,72], actuators [38,73,74,75] and batteries [45,68,69,70,72,76,77]. This review mainly focuses on the recent progress of LIG based flexible electronics, including the mechanical sensors monitoring the motion of the human body, the temperature or humidity sensors monitoring the environmental changes, the electrochemical electrodes connecting the electrical components, and the heaters that can control the bio-inspired actuators. The research progress in related fields is summarized through the introduction of these devices. Finally, we provide some perspectives on the remaining challenges and opportunities of LIG in terms of preparations and practical applications.

## 2. Fabrications of LIG

**Figure 1:**
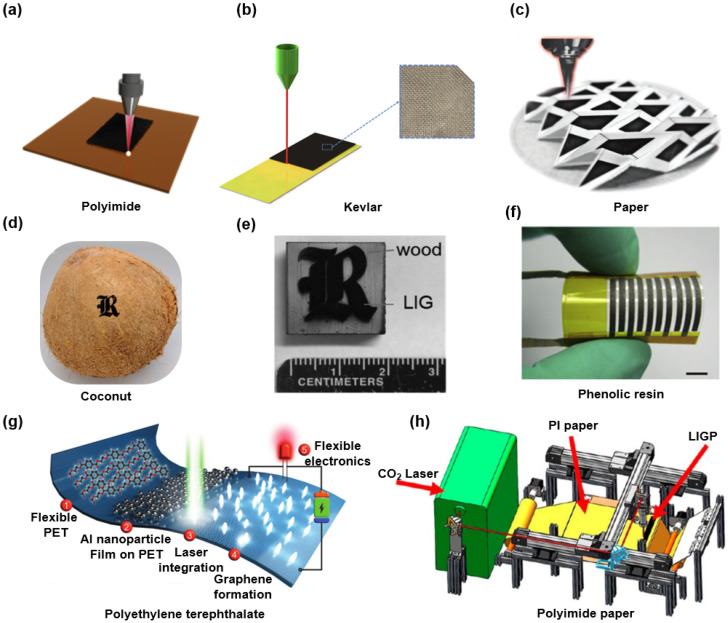
Fabrication of LIG on diverse substrates. (**a**) Polyimide film. Reprinted with permission from Ref. [13], Copyright 2018, Elsevier B.V. (**b**) Aramid fiber fabric. Reprinted with permission from Ref. [27], Copyright 2021, Elsevier B.V. (**c**) Paper soaked with the gelatin-mediated inks containing molybdenum ions. Reprinted with permission from Ref. [30], Copyright 2018, Wiley-VCH. (**d**) Coconut shell. Reprinted with permission from Ref. [24], Copyright 2018, American Chemical Society. (**e**) Dry wood. Reprinted with permission from Ref. [22], Copyright 2019, Wiley-VCH. (**f**) Phenolic film. Reprinted with permission from Ref. [32], Copyright 2018, Elsevier B.V. (**g**) Polyethylene terephthalate/Al nanoparticle composite. Reprinted with permission from Ref. [31], Copyright 2021, Wiley-VCH. (**h**) Polyimide paper. Reprinted with permission from Ref. [28], Copyright 2020, American Chemical Society.

Since the Tour’s group first prepared porous graphene films with three-dimensional networks from commercial polymer films using a CO_2_ infrared laser [10]. The PI thin film is still the most common carbonaceous substrate for LIG. The sharp rise of the localized temperature into the PI substrate due to the laser irradiation breaks the C–O, C=O, and N–O bonds and leads to the recombination of the C–C bond. In other words, the sp3-carbon atoms are photo-thermally converted to sp2-carbon atoms by pulsed laser irradiation. The formation of porous graphene morphology is due to rapid outgassing from the PI melt. This laser inducted carbonation process can be divided into four stages: PI sheets, sheet nanostructure breaking into fibers, graphitization occurs in plates and fibers, and transition from fiber to carbonized droplets [13]. Numerous efforts have been devoted toward experimentally analyzing the effects of the manufacturing parameters on the morphology and electrical properties of LIG on PI films. They suggest that laser wavelength, output power, focal length, and pulse distribution all affect the performances of LIG [10,13,24,27,35,36]. The LIG with desired morphology and resistance can be prepared purposefully for its various design parameters. Additionally, the LIG has also been successfully realized on multiple natural and synthetic materials. As shown in Figure 1, diverse substrates such as plants (i.e., woods, leaves, potato skins and coconut shells) [22,23,24], fabrics (i.e., Kevlar and silk) [24,25,26,27], papers (i.e., printing paper and PI paper) [28,29,30], and polymers (i.e., polyethylene terephthalate and phenolic resin) are transformed into graphene directly by laser irradiation [17,27,31,32,33,34]. The exceptional design ability in the electrical performances and the wide selection of carbon precursors demonstrate the potential of LIG in the fields such as flexible, large-scale, and biodegradable electronics.

## 3. Application of LIG

**Figure 2:**
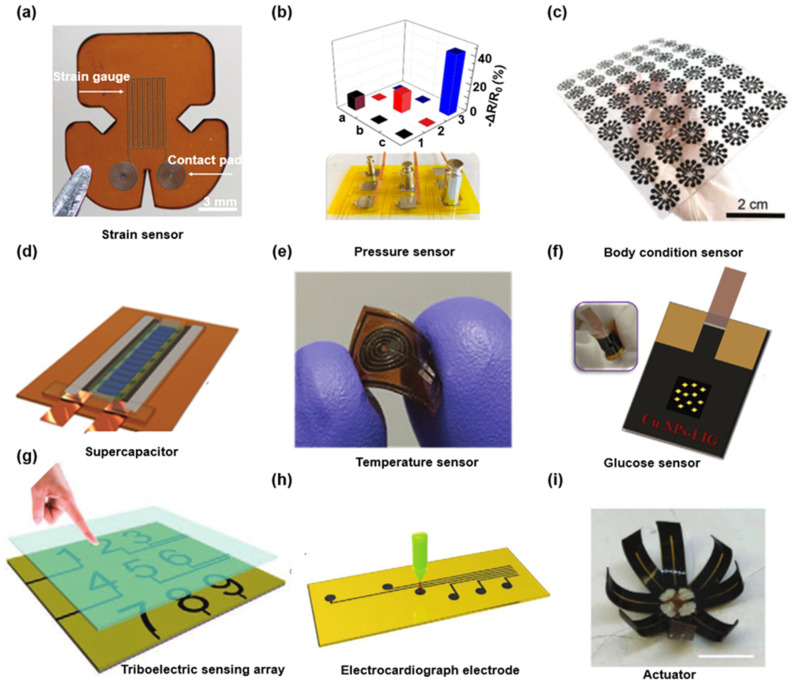
Various functional LIG flexible electronic devices. (**a**) LIG-based strain gauges produced by UV laser. Reprinted with permission from Ref. [18], Copyright 2018, Wiley-VCH. (**b**) MWCNTs-LIG asymmetric pressure sensor array. Reprinted with permission from Ref. [79], Copyright 2021, Elsevier B.V. (**c**) A large-scale LIG-based core body arrays for the tilt sensor. Reprinted with permission from Ref. [80], Copyright 2021, Wiley-VCH. (**d**) LIG-based pseudocapacitive micro-supercapacitors. Reprinted with permission from Ref. [49], Copyright 2015, Wiley-VCH. (**e**) LIG-based highly linear and stable flexible temperature sensor. Reprinted with permission from Ref. [81], Copyright 2020, Wiley-VCH. (**f**) Non-enzymatic LIG-based glucose sensors. Reprinted with permission from Ref. [82], Copyright 2020, Elsevier B.V. (**g**) Nine-digital arrayed numeric touch panel based on the flexible high-resolution triboelectric sensor array. Reprinted with permission from Ref. [58], Copyright 2021, Wiley-VCH. (**h**) LIG-based dry electrode for electrocardiogram monitoring. Reprinted with permission from Ref. [85], Copyright 2021, Wiley-VCH. (**i**) LIG-based 3D flower-like actuator. Scale bar: 1 cm. Reprinted with permission from Ref. [73], Copyright 2020, Wiley-VCH.

Since the LIG-based capacitor was first realized in 2014 [10], improved precursor materials and structural designs have been explored to prepare LIG and LIG-based devices with controlled morphologies and tailored properties. This section mainly overviews the LIG fabricated on various carbonaceous substrates and diverse applications of LIG-based devices. By introducing these outstanding research results, the multi-functional applications of LIG ascribing to its unique advantages of a high degree of designing freedom is demonstrated. Figure 2 depicts the applications of LIG-based electrical systems in different fields, ascribing to its free-designability, the multi-substrate adaptation, and excellent electrical properties. Figure 2a illustrates the LIG-based strain gauges with a high sensitive and wide tunable range under the irradiation of the ultraviolet laser [18]. Compared with previously reported LIG formed under the irradiation of the infrared laser, LIG prepared by ultraviolet laser has a smaller minimum line-width down to 50 µm than that achieved by CO_2_ infrared lasers (i.e., 100 µm) [78]. The stable and high-quality radial pulse and carotid pulse rate signals were recorded immediately by this LIG-based strain gauges after adhered onto the wrist and neck of a volunteer. Figure 2b gives a novel flexible asymmetric pressure sensor composed of multi-walled carbon nanotubes (MWCNTs) and LIG [79]. Benefiting from the microstructure of 3D porous LIG nanosheets, the pressure sensor exhibits some specific features such as ultra-sensitivity (i.e., minimum to 1.2 Pa), super rapid response recovery (i.e., about 2 ms), and good durability (i.e., >2000 cycles). And the high-performance pressure sensors can detect various subtle human motions (i.e., breath, vocal vibration, and wrist pulse) in real-time. Additionally, the integrated pressure sensor array can simultaneously monitor multiple points with varying degrees of applied pressure. Figure 2c shows an exquisite design, a versatile LIG-based integrated, flexible sensor system, which can wirelessly monitor the sleeping postures, respiration rate, and humidity of the diaper moisture [80]. The unique tilt sensor takes advantage of the super-hydrophobic surface of LIG. Notably, the liquid metal that can roll freely on its surface connects different LIG circuits to identify the tilt direction according to different tilt angles. The tilt sensor confining a liquid metal droplet inside a cavity can track at least 18 slanting orientations. The earliest application of the LIG-PI system as a supercapacitor is realized by Tour’s group [10]. On this basis, as shown in Figure 2d they have improved the micro-supercapacitors (MSC) with interdigitated electrodes using a hybrid composite of LIG [49]. A highly stable and linear LIG-based flexible temperature sensor capable of delivering the real-time monitor of human skin temperature was shown in Figure 2e [81]. Benefiting from the fast electron transfer between the Cu nanoparticles and the porous graphene, a novel LIG-based glucose biosensor provides a new platform for the fabrication of the flexible non-enzymatic glucose sensors (as shown in Figure 2f) [82]. Additionally, the LIG represents a class of triboelectric materials that can be used as a triboelectric generator (TENG) converting mechanical energy into electrical energy. As a passive electronic device that bypasses the limitation of power supply or battery, TENG shows an exceptional promise in the wireless device and the wearable sensing electronic [54,55,57,59]. As shown in Figure 2g, the flexible triboelectric sensing array comprises a touch panel with 9-digital channels based on a LIG-patterned TENG [58,60]. To bypass the constraints from the underlying robustness polymer substrate and the delamination by wear and tear [83,84,85,86,87,88,89,90], LIG was transferred from the substrate by polydimethylsiloxane (PDMS). Notably, the LIG adhered onto the soft and stretchable PDMS film yields flexible, durable, and high-performance electrocardiography monitoring dry electrodes (as shown in Figure 2h) [85]. Figure 2i gives the demonstration (i.e., a LIG-based 3D flower) of the LIG actuator composed of a PI thin film and LIG/PDMS coating. Herein, the LIG serves as a flexible heater to increase the temperate via joule-heating. The thermal strain mismatch between PI and PDMS can induce bending deformations of the actuators toward the PI side [73].

### 3.1. LIG-Based Biosensors

Recently, flexible electronic devices are becoming increasingly important for the urgent demands of motion capture, sports rehabilitation, and health monitoring [91]. These electronic devices have an elastic modulus similar to skin [92,93] and can be intimately adhered to the human organs (e.g., wrist) for continuous and real-time data acquisition [42,83,90], thus dynamically picturing the body’s states and environmental conditions. Since its formation through a laser-assisted, large-area, low-cost, facile, efficient, and anon-mask technology on the versatile substrates, the LIG has attracted increasing attention in serving as a conductive or functional material in flexible electronics. Furthermore, the microstructure and the electrical conductivity of the prepared porous graphene films can also be preciously designed by controlling the laser-scanning parameters. At present, various types of LIG-based electronic devices have been successfully proposed to monitor mechanical [16,18,37,58,78,79,83,84,87,88,89,90,94,95,96], environmental [80,81,84,97,98,99,100,101,102] and biochemical signals [19,67,82,85,90,103,104,105,106,107].

#### 3.1.1. LIG-Based Mechanical Sensors

**Figure 3:**
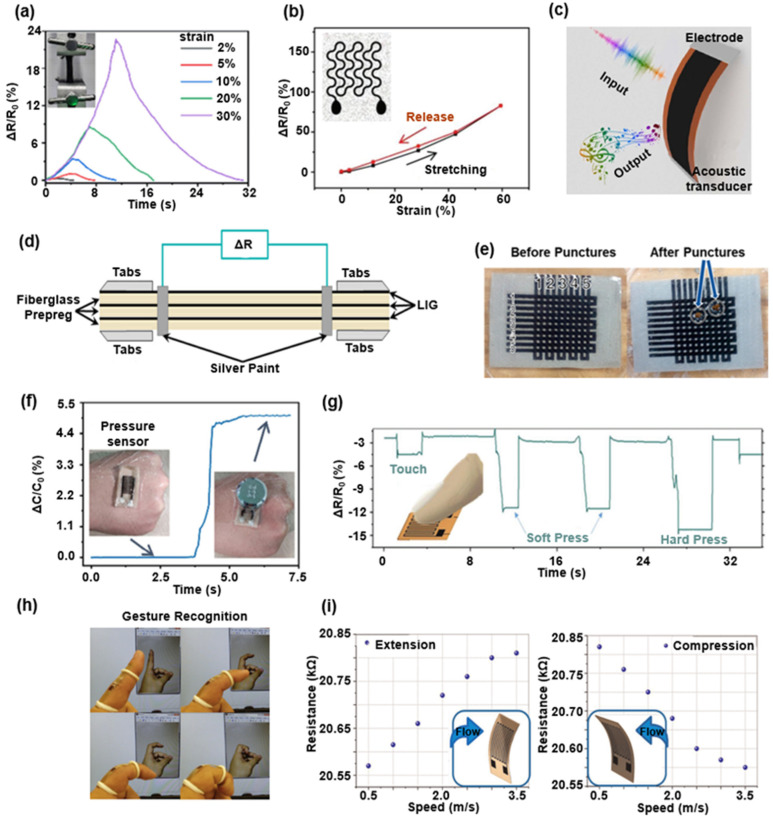
Various LIG-based mechanical sensors. (**a**) The normalized resistance of the LIG-based waterproof wearable sensors versus the various tensile strains at a fixed strain rate. Reprinted with permission from Ref. [84], Copyright 2018, Wiley-VCH. (**b**) The normalized resistance variation of LIG-based gas-permeable on-skin sensor in a typical stretch/release cycle. Reprinted with permission from Ref. [83], Copyright 2018, Wiley-VCH. (**c**) LIG-based artificial throat with the ability of emitting and detecting sound. Reprinted with permission from Ref. [16], Copyright 2017, Springer Nature. (**d**) Schematic of the prepared LIG-based fiberglass sample for fatigue damage tracking. Reprinted with permission from Ref. [96], Copyright 2021, Elsevier B.V. (**e**) Puncture sensing arrays based on Laminated LIG Composites. Reprinted with permission from Ref. [88], Copyright 2020, American Chemical Society. (**f**) LIG-based waterproof capacitance pressure sensor. Reprinted with permission from Ref. [84], Copyright 2018, Wiley-VCH. (**g**) The relative resistance variation of LIG-based pressure sensor induced by touch from the index finger. Reprinted with permission from Ref. [37], Copyright 2020, Wiley-VCH. (**h**) Gesture recognition by LIG-based curvature sensor. Reprinted with permission from Ref. [78], Copyright 2016, Elsevier B.V. (**i**) The response of the LIG-based bending sensor to different flow velocities. Reprinted with permission from Ref. [95], Copyright 2019, Springer Nature.

The LIG-based strain sensor shown in Figure 2a has a monitoring range of 1% strain [18]. Although the LIG can serve as the functional material of the strain sensor with high sensitivity and good stability for its 3D porous microstructure and the piezoresistive effect, the limitations from its in-plane robustness impose certain constraints on its applications in the fields that require a high-level stretchability (e.g., >30%). Transferring the LIG from the PI substrate onto the stretchable elastic substrate (e.g., PDMS) will significantly enlarge the accessible strain range of the flexible strain sensor and have demonstrated its advantages in monitoring the multiple motions of human joints. Figure 3a,b depicts the variation of the resistance of two transferred LIG-based sensors as the applied strain increases. The substrate in Figure 3a is a two-component composed silicone with a good durability, biocompatibility, and stretchability [84], and the substrate illustrated in Figure 3b is the silicone elastomer sponges prepared by the sugar template method with high water-vapor permeability [83]. Benefiting from its excellent sensitivity of the porous graphene microstructure, the LIG-based sensor even can detect the infinitesimal deformation caused by small vibrations. The artificial throat is an intelligent LIG-based device that irradiates a 450 nm laser on the PI substrate. As illustrated in Figure 3c, the artificial throat has the integrated functions of emitting and detecting sounds [16]. The underlying mechanism of the LIG-based sound detector lies in minor resistance variations in response to minute air vibrations. In contrast, the LIG-based sound generation relies on the periodic joule-heating generated by alternating current voltages, which cause air expansion and therefore sound wave generation. Figure 3d shows a LIG-based sensor integrated into fiberglass-reinforced composites for in situ monitoring the fatigue damage [87,96]. In this demonstration, the LIG is transferred from the PI film to the surface of an uncured fiberglass prepreg. The fiber-reinforced composites with high specific stiffness are suitable for preparing the sports rehabilitation equipment. Thus, this kind of integrated LIG-based sensor can predict the lifespan of the composite material. The roll-to-roll processing technology has demonstrated its capability in preparing the multifunctional, robust, multilayered, and patterned LIG composites in an exceptional in-plane dimension [28,29,88]. With the aid of this technique, LIG is first transferred onto the thermoplastic films and then patterned into puncture sensing arrays (as shown in Figure 3e). According to the underlying mechanism, pressure sensors based on LIG can be categorized into capacitive and resistive detecting devices. The pressure sensor array shown in Figure 2b utilizes the piezoresistive properties of LIG [79]. In addition, Figure 3f shows a LIG-based capacitive pressure sensor with a cross-finger electrode. In this demonstration, LIG on silicone substrates serves as the electrodes, and the ion gel with ultrahigh capacitance is introduced as the sensing component of the LIG-based pressure sensor. The relative capacitance change (ΔC/C0) of the LIG-based capacitive sensor reaches 500% when a 50 g weight is placed onto the sensor surface [84]. To isolate it from the surrounding environments, the LIG-based resistive pressure sensors composed of PI film with the LIG graphic pattern are encapsulated by a protective polymethyl methacrylate coating. As shown in Figure 3g, the high sensitivity of this sensor allows its potential as a tactile sensor [37]. The LIG-based mechanical sensor can accurately feedback the deformation of the substrate by the bending direction and the degree. Figure 3h illustrates that the LIG-based sensors mounted on the joints of the hand can monitor the bending motion of fingers at different frequencies and angles [78]. Furthermore, the speed and direction of the flow can be monitored by using the LIG-based curvature sensor for its high sensitivity. According to the beam deformation theory, when bending deformations of the sensor is toward the side with LIG, the 3D porous graphene structures compress and contact each other, resulting in reduced resistance. By contrast, the broken connection between the inner porous graphene microstructures results in the continuous increase of the resistance when LIG appears on the outer side of the bending deformation. Thus the direction and the speed of the flow can be characterized by measuring the resistance variation of the LIG-based mechanical sensors, as shown in Figure 3i [95].

#### 3.1.2. LIG-Based Temperature and Humidity Sensors

**Figure 4:**
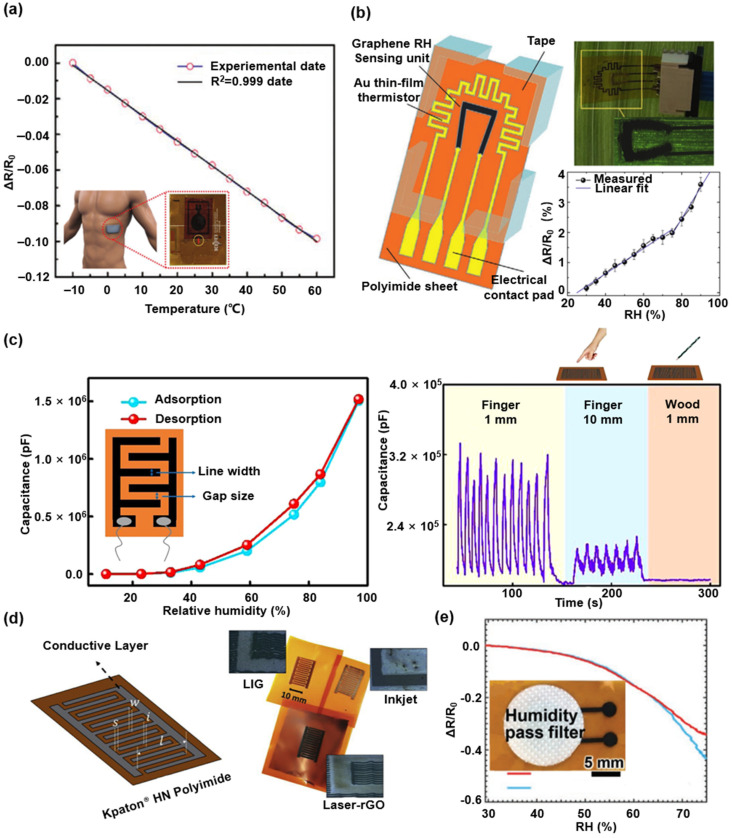
Various LIG-based temperature and humidity sensors. (**a**) Normalized resistance variation of LIG-based temperature sensor versus different temperatures (with interval of 5 °C). Reprinted with permission from Ref. [81], Copyright 2020, Wiley-VCH. (**b**) Normalized resistance variation of the LIG-based RH sensing unit responding to changing environmental RH. Reprinted with permission from Ref. [99], Copyright 2018, Wiley-VCH. (**c**) The capacitance variation of LIG-based RH sensor in a typical adsorption-desorption processes and non-contact object sensing. Reprinted with permission from Ref. [98], Copyright 2020, Elsevier B.V. (**d**) The humidity sensors with various functional conductive materials. Reprinted with permission from Ref. [101], Copyright 2019, Elsevier B.V. (**e**) LIG-based humidity sensor laminated with a water-resistant. Reprinted with permission from Ref. [80], Copyright 2021, Wiley-VCH.

Electrical conductivity changes caused by the electron-phonon scattering and thermal velocity of electrons in response to temperature variations can be adopted for developing LIG-based temperature sensors [81]. As shown in Figure 4a, an integrated, flexible wireless temperature sensing system is proposed with a high linearity LIG-based temperature sensor. Figure 4b shows an integrated temperature and humidity sensor that can be conformably attached to a leaf surface [99]. This flexible electronic device consists of the PI films as the substrate, a LIG-based sensing unit as a humidity (RH) sensor, and an Au-based thin-film thermistor as a temperature sensor. Figure 4c presents a capacitive humidity sensor composed of LIG interdigital electrode and the GO solution coated on LIG electrodes as the sensitive layer [98]. This sensor monitors the variation of the surrounding humidity in a wide range. It distinguishes the type and the distance of different objects approaching the sensor in a non-contact method. In comparation to other functional conductive materials of the capacitive humidity sensors, the LIG-based sensor presents a higher linearity response to the humidity change and a more remarkable thermal stability (as shown in Figure 4d) [101]. Comparatively, the sensitivity of LIG-based sensors shows a slight decrease than the ones involving the laser-reduction graphene oxide and inkjet-printing LIG material. Figure 4e presents a LIG-based humidity sensor laminated with a water-resistant to prevent the sensor from scratches, contamination, and direct moisture contact. The results show that the noncontact detection capability of the LIG-based humidity sensor designed by this method can effectively maintain stable performance under high humidity conditions [80].

#### 3.1.3. LIG-Based Electrochemical Sensors

**Figure 5:**
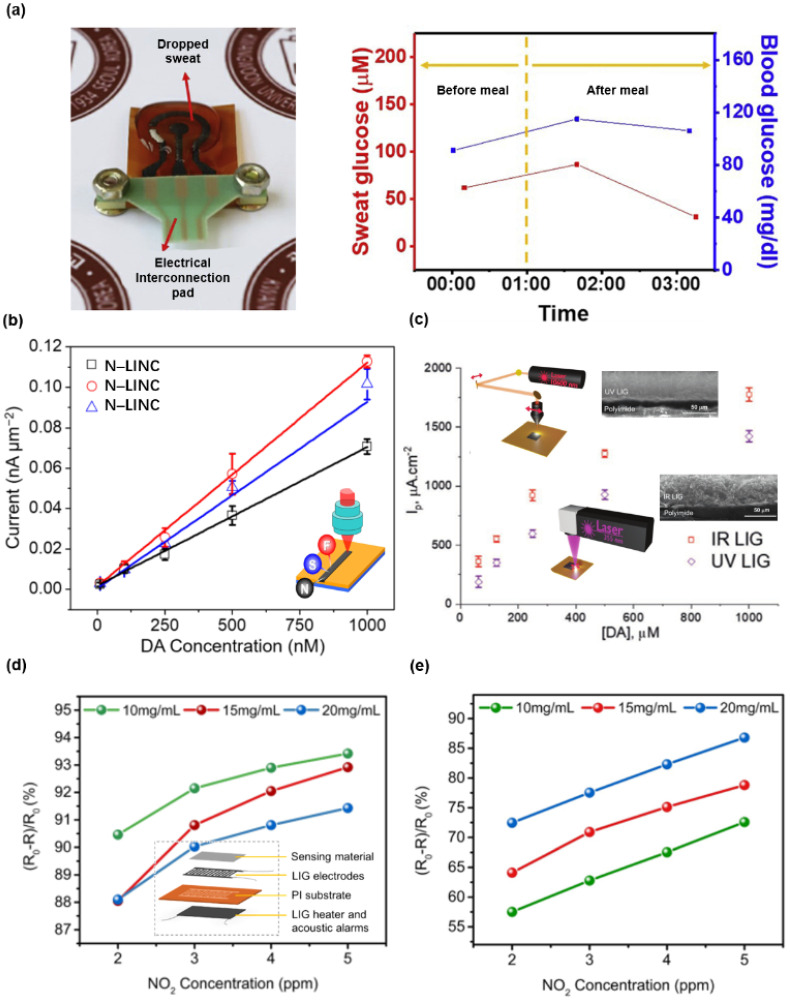
Various LIG-based electrochemical sensors. (**a**) A chemically modified LIG-based flexible ultrasensitive electrochemical glucose sensor. Reprinted with permission from Ref. [106], Copyright 2021, Elsevier B.V. (**b**) Heteroatom-doped LIG-based electrochemical dopamine sensors. Reprinted with permission from Ref. [107], Copyright 2021, Elsevier B.V. (**c**) IR and UV LIG-based dopamine electrochemical sensors. Reprinted with permission from Ref. [19], Copyright 2021, Wiley-VCH. (**d**) The performance of LIG-based NO_2_ sensors at room temperature. (**e**) The performance of LIG-based NO_2_ sensors with Joule-heating at 150 °C. Reprinted with permission from Ref. [67], Copyright 2021, Elsevier B.V.

LIG-based electrochemical sensors show their potential in detecting the contents of organic (i.e., glucose and dopamine) and inorganic matter (i.e., NO_2_ and H_2_O_2_) [9,11,24,26,28,66,69,74,84,85,86,87,88]. As shown in Figure 5a, the sensor was introduced to measure the blood glucose (BG) and the sweat glucose (SG) concentrations before and after meal [106]. The performances of the LIG-based biosensor involving three electrodes for sweat glucose detection are improved by a simple acetic acid treatment, which dramatically increases the ratio of C–C bonds. Dopamine, a neurotransmitter, is a crucial factor for the clinical diagnosis for its function in sending the impulses of cells. As shown in Figure 5b, LIG-based electrically conductive microelectrodes with efficient utilization of heteroatoms exhibit superior performance for electrochemical sensing of dopamine [107]. Figure 5c depicts the comparison of the capabilities of the LIG-based sensors prepared by infrared and ultraviolet laser in detecting dopamine concentration [19] and gives that the infrared LIG-based sensor shows a higher sensitivity. In contrast, ultraviolet LIG is beneficial to miniaturize electrical devices. Figure 5d,e presents a multi-functional NO_2_ monitor and self-alarm device composed of LIG heater, LIG electrodes, and the MoS_2_-based gas sensor [67]. The LIG-based heater provides a stable operating environment of 150 °C, which significantly improves the linearity and sensitivity of the resistance signal compared to the gas sensor’s performance at room temperature. More specially, the LIG heater will be an alarm device when the electrical signal of the sensor is over the threshold value (i.e., NO_2_ concentration is exceeding safety levels). The voltage source connected to the heater is switched to AC, and the alarm is issued.

#### 3.1.4. LIG-Based Electrophysiological Sensors

**Figure 6:**
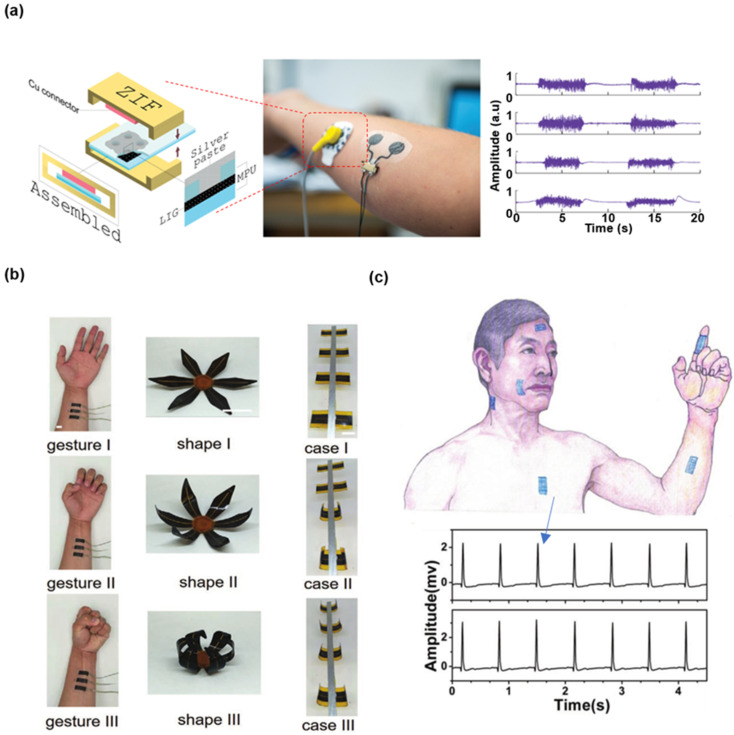
Various LIG-based electrophysiological sensors. (**a**) LIG-F/MPU electrodes on forearm and corresponding EMG recordings over 72 h. Reprinted with permission from Ref. [90], Copyright 2018, American Chemical Society. (**b**) Actuator control of the LIG-based EMG sensors. Scale bars: 1 cm. Reprinted with permission from Ref. [73], Copyright 2018, Wiley-VCH. (**c**) ECG signals measured from the LIG-based sensors and conventional gel electrodes. Reprinted with permission from Ref. [83], Copyright 2018, Wiley-VCH.

When the cell is stimulated and excited by the external factors, the membrane potential of the stimulated place will produce a series of transient changes, initially the membrane potential increases and is then slowly restored to rest potential, the transformation of this membrane potential is an electrophysiological signal. Some LIG-based flexible electrophysiological sensors have been proposed and can be attached on various parts of human bodies to record corresponding electrophysiological signals. As shown in Figure 6a, a surface electrophysiological electrode was consisted of embedding LIG fibers (LIG-F) into a thin and soft medical grade polyurethane (MPU). In addition, the LIG-based electrode was strengthened by a soft vertical interconnect access (VIA) connector design which enabled wiring from the top of the composite and not from the bottom. The electrodes reliably worked on the forearm of a volunteer for three consecutive days, during which the volunteer kept to his routine, including exercise and bathing. After 72 h, the obvious increase of signal noise indicated the electrode failure [90]. Figure 6b presents the LIG-based electromyogram (EMG) sensors closely adhered onto the wrist of the volunteer (muscle: flexor carpi radialis) to record the electromyographic signals. The LIG-based actuator can be remotely controlled by the human motion signals recorded by the LIG-based sensor [73]. As illustrated in Figure 6c, the gas-permeable on-skin electrophysiological sensors made of the porous materials exhibit outstanding capabilities in recording electrophysiological activities from the skin [83]. Electrocardiogram (ECG) signals were measured from the chest of a volunteer with the LIG-based sensors (top ECG) and conventional gel electrodes (bottom ECG), respectively. The high-quality signals illustrate the LIG-based sensor’s excellent performance compared to that of the commercial products.

### 3.2. LIG-Based Energy Storage Devices

With the rapid development of the wireless highly-integrated flexible electronic, energy storage devices with good flexibility and high energy density are urgently demanded. Benefiting from the high conductivity and binder-free self-supported microstructure, LIG has shown promising applications in miniaturized energy storage devices. Recent studies have revealed its capability in serving as the current collector for lithium-metal batteries, catalysts for metal-air batteries, and electrodes for supercapacitors. 

#### 3.2.1. LIG-Based Batteries

**Figure 7:**
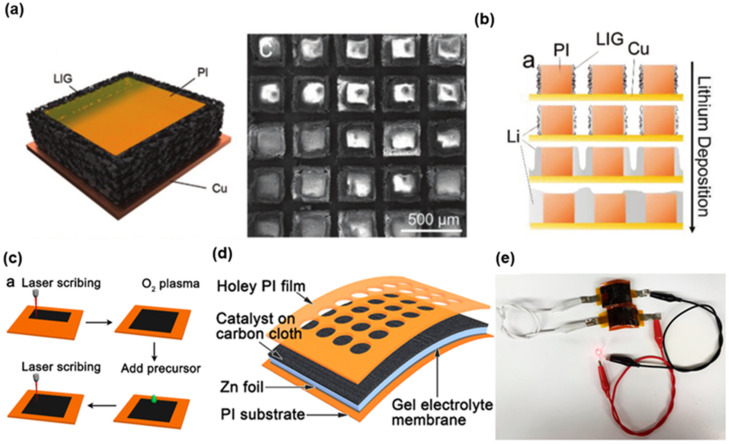
Various LIG-based batteries. (**a**) Illustration and SEM image of the microstructure of LIGHS@Cu. (**b**) Illustration of the growth mode of lithium in LIGHS@Cu. Reprinted with permission from Ref. [77]. Copyright 2019, Wiley-VCH. (**c**) Schematic fabrication process of the LIG-based catalysts. (**d**,**e**) Schematic structure and practical demonstration of LIG-based hybrid catalysts for Zn-Air battery. Reprinted with permission from Ref. [69], Copyright 2019, American Chemical Society.

The carbon nanomaterial of LIG has the combined capability of high porosity, high electrochemical stability, and relatively good electron conductivity. Additionally, the large surface area and the good electron conductivity of LIG coincide with the essential requirements of ideal electrode material. Figure 7a presents a 3D-hierarchical composite material consisting of copper-PI substrate and LIG array [77]. Compared to the copper foil, the numerous defects and heteroatoms present in LIG hierarchical structure on copper foil (LIGHS@Cu) significantly lower the Li nucleation barrier, and the full lithium-metal battery based on LiFePO4 (LFP) cathode with LIG composite current collector extended cycle life for over 250 cycles from 50 cycles. Figure 7b illustrates the growth mode of lithium in LIG composite. During the charging process, the lithium starts nucleation in the LIG around the PI pillar, instead of on the exposed Cu surface. Then, the lithium deposition thickens the lithium layer until it covers the entire surface of the entire region. In particular, Tour’s group has obtained considerable achievements in the research of utilizing LIG as hybrid catalysts for metal-air batteries [69,70,72,108]. As shown in Figure 7c, the ternary metal oxide/LIG hybrid catalysts were directly formed by a CO_2_ laser. The porous microstructure of LIG contributes to a good carrier to anchor the metal nanoparticles. The porous and interconnected structure enables a high active surface area that contributes to good contact with the electrolyte. Figure 7d,e presents the schematic structure and application of the flexible Zn-air battery with a LIG-based cathode catalyst. A single battery can provide an open-circuit potential of 1.35 V and the serial connection of two batteries were capable of powering a LED [69].

#### 3.2.2. LIG-Based Supercapacitors

**Figure 8:**
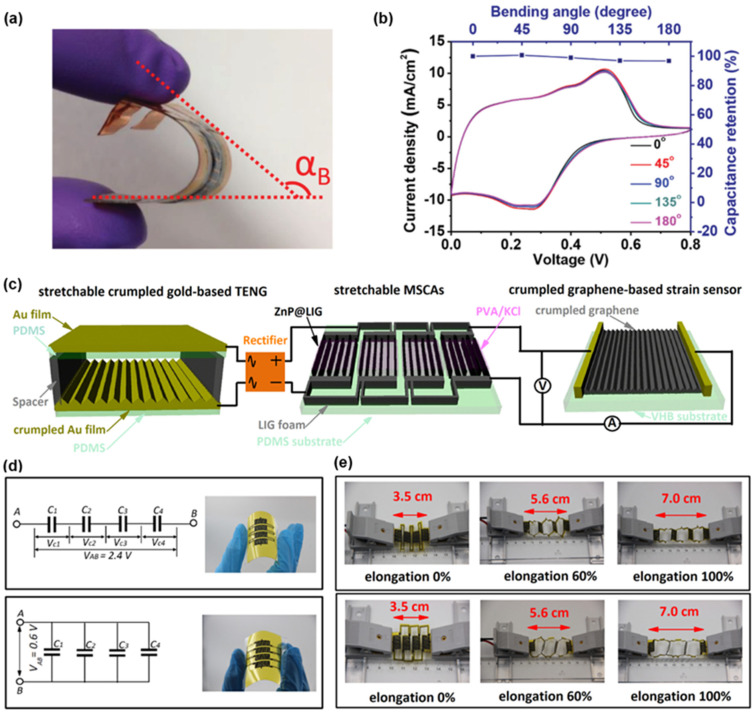
Various LIG-based supercapacitors. (**a**) Digital photograph of flexible MSC device. (**b**) Flexibility testing of LIG-PANI MSC. Reprinted with permission from Ref. [49], Copyright 2020, Wiley-VCH. (**c**) Schematic of the stretchable LIG-based MSCAs charged by the crumpled Au-based TENG to power the stretchable crumpled graphene-based strain sensor. (**d**) The equivalent circuit diagrams and photographs of the four MSC devices connected in series and parallel. (**e**) Real-time images of all-in-one stretchable MSCAs comprised of four devices interconnected in series and parallel configurations upon a tensile strain up to 100%. Reprinted with permission from Ref. [54], Copyright 2020, Elsevier B.V.

The supercapacitor is a new type of energy storage device between traditional capacitor and rechargeable battery. The most significant advantages of supercapacitor are the long cycle life, the fast charging rate, and the high power density. Compared to the sandwich structure, the supercapacitors with interdigitated coplanar electrode architecture exhibit better rate capability and higher power density. LIG is considered as a potential candidate material for supercapacitors for its high-resolution patterns and excellent electrical conductivity. Figure 8a presents a flexible LIG-based micro-supercapacitor (MSC). The preparation of interdigitated electrode began with the formation of LIG onto PI thin film by the CO_2_ laser. Then the electrodeposition of pseudocapacitive materials on LIG was achieved with a three-electrode setup. As shown in Figure 8b, the MSC devices demonstrate mechanical flexibility and stability without sacrificing their capacitance retention [49]. Figure 8c presents a self-powered stretchable system consisting of all in-one LIG-based micro-supercapacitor arrays (MSCAs), a crumpled Au-based TENG, and a crumpled graphene-based strain sensor. The all-in-one MSCAs are based on non-layered ZnP ultrathin nanosheets anchored on the 3D porous LIG, which is configured in the islands-bridge layout. The equivalent circuit and optical images of MSCAs are shown in Figure 8d. The output voltages of the four LIG-based MSC devices connected in series and parallel are 0.6 and 2.4 V, respectively. The MSCAs with islands-bridge structure can withstand a large mechanical deformation (i.e., up to 100% applied strain), meanwhile the capacitance retention is almost constant throughout the tensile deformation process. When the tensile strain is 100%, the capacitance retention decreases by 2.5% and 2.8% in serial and parallel connection modes, respectively. Figure 8e demonstrates the images of MSCAs connected in series and parallel at different tensile strain stages from 0% to 100% [54].

### 3.3. LIG-Based Heaters and Actuators

**Figure 9:**
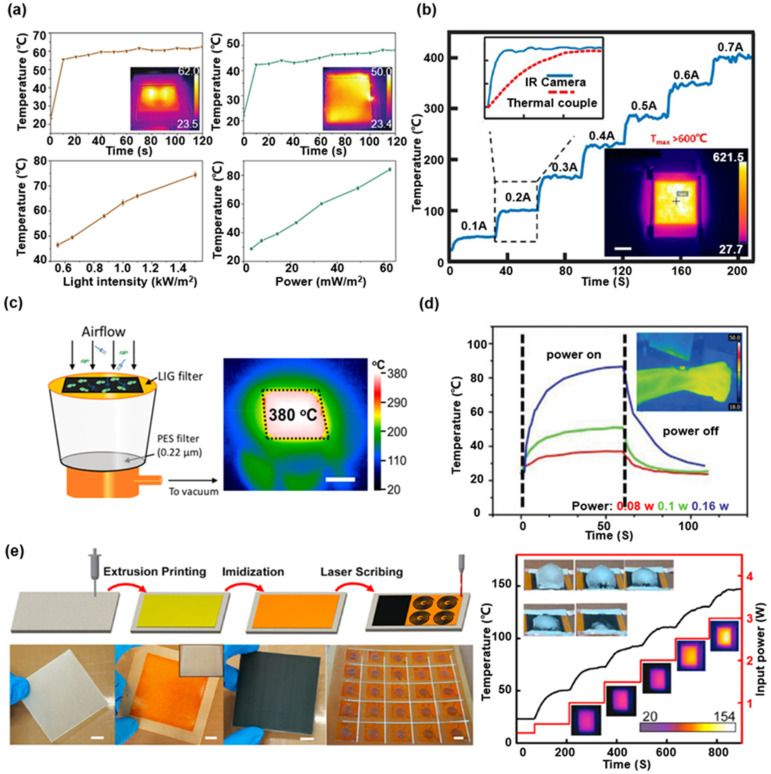
Various LIG-based heaters. (**a**) Photothermal effect and Joule heating effect of hydrophobic LIG-based heater. Reprinted with permission from Ref. [65], Copyright 2021, Wiley-VCH. (**b**) Heating properties of LIG-based PI paper heaters in ambient air. Reprinted with permission from Ref. [28], Copyright 2020, American Chemical Society. (**c**) Self-sterilizing LIG-based air filter. Reprinted with permission from Ref. [62], Copyright 2019, American Chemical Society. (**d**) Temperature responses of the LIG-based gas-permeable on-skin joule-heating patch at various incident powers. Reprinted with permission from Ref. [83]. Copyright 2018, Wiley-VCH. (**e**) The preparation, Joule-heating performance and de-icing demonstration of LIG-based composite. Reprinted with permission from Ref. [64], Copyright 2021, Elsevier B.V.

Benefiting from its unique Joule-heating and hydrophobic properties, LIG is considered as a potential candidate material for the antibacterial [62,65], gas sensing [66,67] and de-icing applications [64]. The heating curves and thermal images of LIG under simulated Xenon sunlight (i.e., 1 kW·m^−2^) and DC voltage (i.e., 7.5 V) shown in Figure 9a demonstrate that the LIG based heater can reach a 46 °C change, a sufficient temperature increase for the virus inactivation [65]. Figure 9b presents the LIG-based heater prepared through the large-scale processing on PI paper by the roll-to-roll fabrication technology [28]. In ambient air, the LIG-based heater can gradually increase the temperature, stabilizing by stepwise enhancing the current level from 0.1 to 0.7 A. For a constant input power of 130 W, the infrared thermal image shows a working limit state of LIG-based heater at 600 °C. Benefiting from the high surface area brought by the porous microstructure, excellent joule thermal performance, and thermal stability of LIG. A LIG-based sterilizable air filter is shown in Figure 9c [62]. Through a periodic Joule-heating, the thermally stable LIG-based filter destroys a variety of pathogenic microorganisms in filtering the air. In addition to sterilizing and destroying viruses, the on-skin LIG-based joule-heating patches are helpful for biomedical treatment, as shown in Figure 9d. These LIG-based sugar-templated silicone elastomer sponge devices exhibit high water-vapor permeability, 18 times higher than the silicone elastomers without pores. As an on-skin heater, good permeability promotes evaporation of sweat during use, and minimizes discomfort and inflammation risks [83]. As shown in Figure 9e, the subfigure on the left illustrates the imidization process of polyamic acid coating as the substrates for preparing LIG. To utilize the Joule-heating performance, a de-icing application was demonstrated by a LIG-based heater, where a super-hydrophobic surface of LIG effectively prevents residue accumulation throughout the process [64].

**Figure 10:**
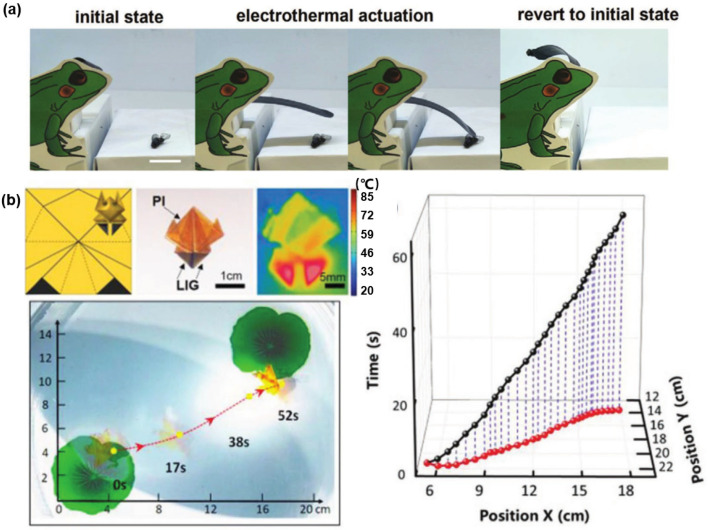
Various LIG-based actuators. (**a**) A biomimetic frog tongue consist of LIG-based actuator. Scale bars: 1 cm. Reprinted with permission from Ref. [73], Copyright 2020, Wiley-VCH. (**b**) Linear movement of the origami frog actuator with two LIG legs. Reprinted with permission from Ref. [38]. Copyright 2020, Wiley-VCH.

The Joule-heating and photothermal properties of LIG can be used not only in the design of heaters but also in the actuators. In the previous introduction about Figure 2i and Figure 6b, the working mechanism and application of the Joule-heating actuator were mentioned, respectively. Various biomimetic soft structures and flexible electronic devices have become the research hotspots in the field of flexible electronics [109,110]. The main content in this section is the introduction of bio-inspired actuators. As shown in Figure 10a, the biomimetic LIG-based frog tongue rolled up at room temperature. Inside LIG heated the curled actuator under a voltage of 20 V, meanwhile, the LIG-based actuator unbent and elongated to capture a fly by a significant mismatch in thermal expansion between PI and PDMS [73]. As shown in Figure 10b, LIG is reported to develop light-driven actuators based on the Marangoni effect. The PI film with pre-written LIG patterns was folded into an origami frog. Under light irradiation, the localized temperature at the LIG-based legs was much higher than the other regions, and the surface tension around the LIG-based legs decreased more obviously. The LIG-based origami-frog actuator moved forward by the local unbalanced surface tension [38].

## 4. Conclusions and Perspectives

Since the first LIG prepared from commercial PI films in 2014, the technologies related to LIG have developed rapidly. LIG that possesses a good combination of conductivity and flexibility, and laser-assisted manufacturing provides a facile, efficient, and low-cost and customizable approach to making graphene-based electronics. Abundant research results show that LIG has excellent potential in the fields ranging from wearable electronics, health monitors, motion captures to the soft robot. To summarize, this article systematically reviews the properties and typical applications of LIG-based electronic devices in aspects from mechanical sensors, temperature and humidity sensors, electrochemical sensors, electrophysiological sensors, heaters, and actuators. The conductive capability and porous microstructure ascribable to the LIG characters ensure the LIG-based mechanical sensor are more precise and sensitive. The patterning preparation and diverse choices of substrates make LIG a better choice in the wearable biosensors for detecting biological signals ranging from temperature, humidity, glucose dopamine to ECG. The Joule-heating and photothermal properties have shown its potential in the heaters for disinfection or the bio-inspired soft robot actuation.

At present, the most of LIG-based devices are still in the laboratory testing stage. There are still some challenges for researchers to focus on. For example, the minimum width of the irradiated LIG line is about 12 µm under visible fiber-coupled 405 nm laser, 50 µm under ultraviolet laser and 60–100 µm under infrared laser, respectively [78,111]. The resolution of laser technology should be improved by using finely focused laser beams to achieve high resolution electrode patterns. The conditions of the laser scanning procedure (i.e., the laser power, the frequency, the scanning speed and the pulses per inch and focal length) offer great influences on the electrical and morphology of LIG [10,13,24,27,35,36]. Most of the current studies have been focused on uniform stacked graphene structures. Further, as it is possible to control the degree of graphitization, the preparation parameters can be controlled programmatically to obtain modular heterogeneous graphene patterns for some specific demands. LIG-based devices may be damaged due to its fragile porous structure. A novel strategy that can be used to protect LIG without compromising its performance is expected. High integration, wireless transmission and graphical display of singles is also the developing trend of LIG based devices. By rationally merging the LIG-based tilt, strain, and humidity sensors on a thin flexible film, a sensor system with wireless feedback alarm functions will be a good example [65]. LIG can construct complex and fine patterns on flat flexible substrates. Currently, there are a series of studies on 3D self-assembly devices [112,113,114]. In the future, LIG may be used for planar fabrication and then become a new device through 3D self-assembly. To satisfy the future application in a complex practical environment, tremendous efforts are required to deeply understand its instinct properties and long-term performances. Finding optimal substrates and more appropriate fabricating strategies are vitally crucial for fully exerting their potential in flexible electronics. Predictably, the development of the LIG-based flexible electronics is expected to integrate sensing, detecting, and therapeutic functions in the future.

## Data Availability

Not applicable.
